# Supplementary figures and images for: Role of Long Noncoding RNAs ZlMSTRG.11348 and UeMSTRG.02678 in Temperature-Dependent Culm Swelling in Zizania latifolia
Source: Int J Mol Sci. 2021 Jun 2;22(11):6020. doi: 10.3390/ijms22116020 (PMC8199642; doi:10.3390/ijms22116020)

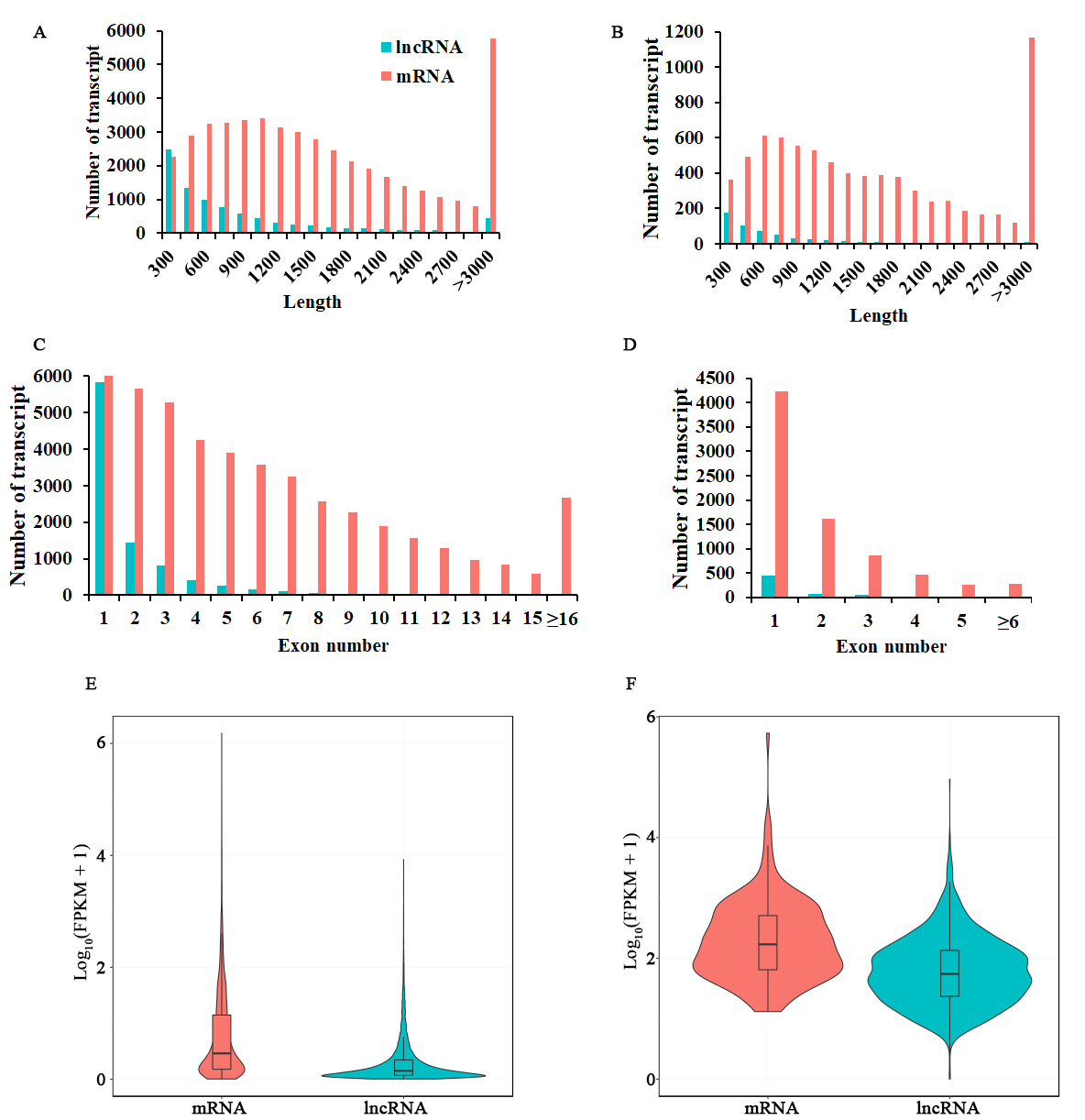

Supplement: Supplementary file 1 [file ijms-22-06020-s001.zip › Figure S1.tif]

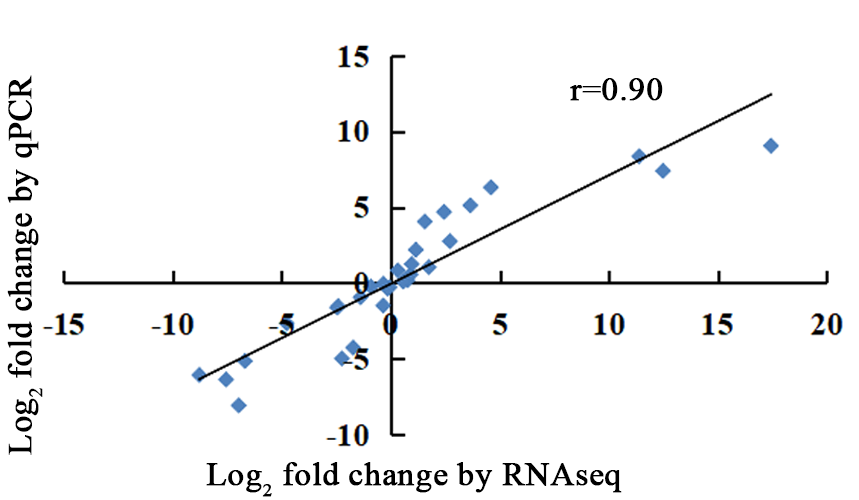

Supplement: Supplementary file 1 [file ijms-22-06020-s001.zip › Figure S2.tif]

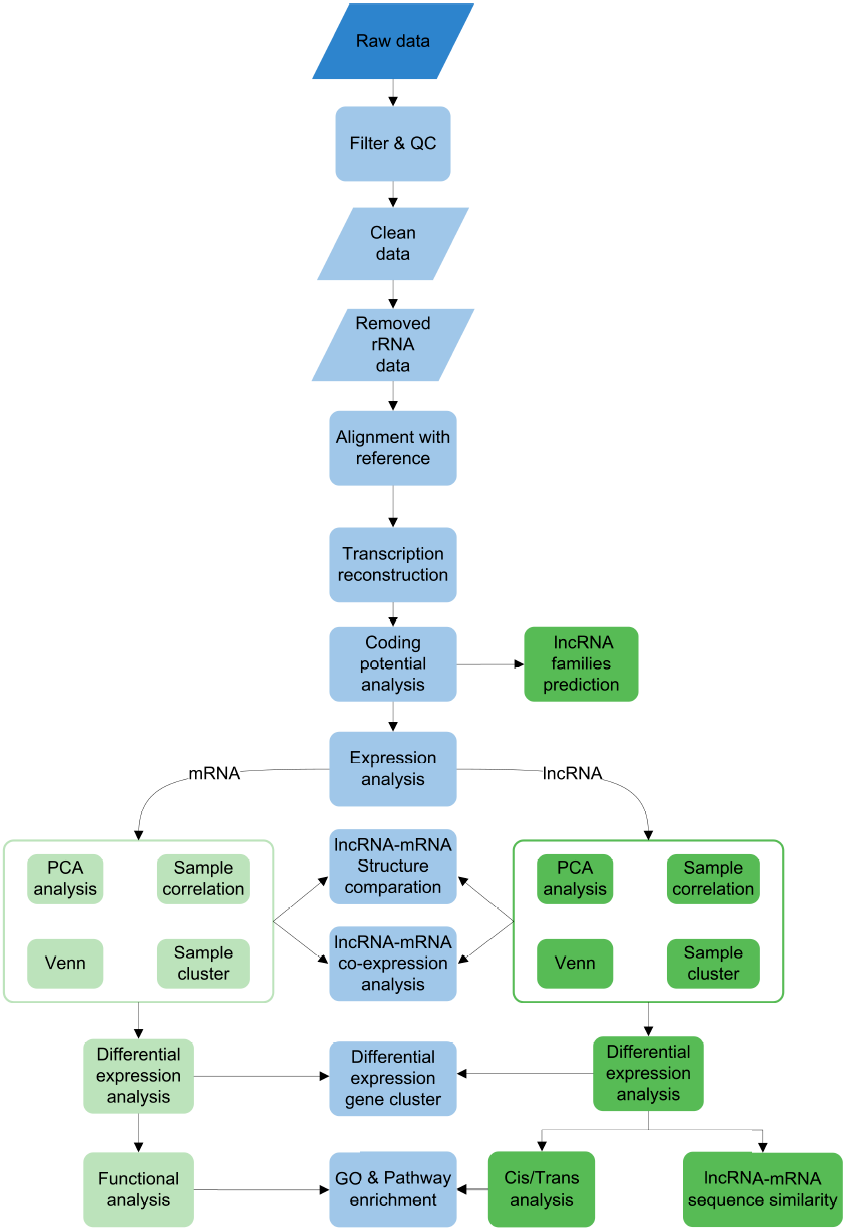

Supplement: Supplementary file 1 [file ijms-22-06020-s001.zip › Figure S3.tif]
